# Knowledge and Attitude of Celiac Disease Among the Population of Riyadh, Saudi Arabia

**DOI:** 10.7759/cureus.68603

**Published:** 2024-09-04

**Authors:** Waleed M Alhuzaim, Omar D AlDawas, Majed Alazmi, Humood AlMutairi, Faisal Altoom, Faris AlShabanat, Belal N Sabbah

**Affiliations:** 1 Department of Internal Medicine, Imam Mohammad bin Saud Islamic University, Riyadh, SAU; 2 Department of Medicine, Imam Mohammad bin Saud Islamic University, Riyadh, SAU; 3 Department of Medicine, AlMaarefa University, Riyadh, SAU; 4 Department of Medicine, Alfaisal University College of Medicine, Riyadh, SAU

**Keywords:** general gastroenterology, knowledge and awareness, riyadh citizens, kingdom of saudi arabia (ksa), celiac disease (cd)

## Abstract

Background and aims

Celiac disease (CD) is a chronic autoimmune disease that is characterized by inflammation of the intestinal mucosa, primarily triggered by gluten. So far, the effective management of CD only includes a gluten-free diet. For early diagnosis and management, adequate knowledge and a positive attitude towards CD are crucial. This study aims to investigate the CD-related knowledge and attitudes of the public in Riyadh, Saudi Arabia.

Methods

A cross-sectional online survey was conducted among individuals aged 16 and older. The data regarding demographic factors, knowledge, and attitudes about CD was collected via an online questionnaire. Statistical analysis was conducted using version 26 of SPSS (IBM Corp., Armonk, NY).

Results

In the current study, 669 individuals responded to the online survey. The majority of participants (82.1%) were familiar with CD. A total of 59.9% of respondents had adequate knowledge, 32.3% had outstanding knowledge, and 7.8% reported no knowledge of CD. The majority (69.5%) of respondents held negative attitudes concerning CD. The correlation between age and CD knowledge (P<0.05) and attitude (P<0.05) was statistically significant. Similarly, the correlation between occupation and CD knowledge (P<0.05) and attitude (P<0.05) was statistically significant. However, no significant association between gender and CD knowledge (p=0.720) or attitude (p=0.244) was found in males and females.

Conclusion

This study revealed that the majority of the residents of Riyadh, Saudi Arabia, had an adequate or excellent understanding of CD. However, the majority of respondents had a negative attitude towards CD management.

## Introduction

Celiac disease (CD) is a chronic immune-mediated enteropathy that is characterized by pronounced gastrointestinal findings. In some cases, extra-intestinal or asymptomatic findings can also be reported [1,2]. This disease can arise from both environmental and genetic factors (human leukocyte antigen). Gluten, an environmental factor is believed to be the major trigger of CD [3]. Furthermore, individuals with autoimmune disease, diabetes, and a family history of CD are more prone to develop CD compared to healthy individuals. CD can cause long-lasting digestive problems and deprive the body of essential nutrients (such as iron, calcium, magnesium, vitamin D, zinc, folate, niacin, vitamin B12, and riboflavin) [4]. The common clinical manifestations of CD in adults include bloating, chronic diarrhea and abdominal pain while in children symptoms such as delayed puberty and failure to thrive are of primary concern [5]. The diagnosis of CD is based on the clinical history of the patient, serological tests, and/or histological findings of the intestine.

In recent times, there has been a significant rise in the prevalence of CD which can be attributed to several factors including greater awareness regarding this disease and utilization of highly sensitive diagnostic tests [6]. Despite that, a significant proportion of patients remain undiagnosed, with reported delay in diagnosis of four to seven years [7]. The number of undiagnosed individuals is even greater in developing countries due to the scarcity of diagnostic tools and trained physicians. Currently, the prevalence of CD is estimated at 0.5%-1% across the world [8]. However, the incidence of CD is a bit higher in Saudi Arabia. A meta-analysis conducted in 2018 reported that the seroprevalence of CD was 2.7% while biopsy-proven CD was 1.4% in Saudi Arabia [9]. Similarly, a cross-sectional study conducted in Saudi Arabia which included a total of 729 children showed that the most common complication in CD is malnutrition (27.8%) and bone weakening (15.5%) [10].

CD can have detrimental effects on the long-term health of children and adults alike. Early diagnosis is crucial for treating CD and avoid long-term complications [11]. One of the reasons for delayed diagnosis can be the lack of knowledge about CD among the general public and primary care physicians. As CD has various systemic ramifications for affected individuals, adequate knowledge of the condition is paramount for effective preventive measures including gluten-free diet. So far, limited studies have been published that tried to assess the knowledge and attitude of the public regarding CD in Saudi Arabia. The present study aimed to determine the knowledge and attitudes of the population of Riyadh, Saudi Arabia toward CD.

## Materials and methods

Study design and settings

This cross-sectional study was conducted to determine the knowledge and attitude of CD among the general public. The study was carried out in Riyadh, Saudi Arabia.

Study population

The study involved both males and females aged over 16 years. The inclusion criteria consisted of being aged over 16 years and being in Riyadh, Saudi Arabia while the exclusion criteria were being under 16 or from outside Riyadh, Saudi Arabia. The sample size for this study was determined using the Roasoft online calculator with a confidence level of 99%, an estimated response rate of 50%, and an error margin of 5%. The minimum required sample size was calculated to be 664, and non-probability convenience sampling was used to acquire the samples.

Data collection

A self-made online questionnaire was utilized to collect data, with questions pertaining to age, gender, occupation, knowledge, and attitude about CD. The questionnaire was distributed via social media in a convenience-based model. The knowledge questions were coded as correct (1) or incorrect or “I do not know” (0), and the scores were added to classify participants as having no knowledge (score of 0), fair knowledge (score of 1 to 6) or excellent knowledge (score of 7 or higher). Similarly, participants were classed as having a negative attitude (score of 0 to 2) or a good attitude (score of 3 or higher). In addition, the questionnaire inquired about participants' awareness of gluten-free items in food markets and restaurants, the difficulties in getting gluten-free products, and the social impact of CD.

Statistical analysis

Version 26 of the Statistical Package for the Social Sciences (SPSS; IBM Corp., Armonk, NY) was used for the statistical analysis of the data collected from the self-designed online questionnaire. To describe the demographic information of the participants, including age, gender, and occupation, descriptive statistics were utilized. For categorical variables, frequencies and percentages were computed, whereas means and standard deviations were computed for continuous variables. Using inferential statistics such as chi-square tests, t-tests, and analysis of variance (ANOVA), the associations between demographic characteristics and celiac disease knowledge and attitude were examined. A p-value less than 0.05 is statistically significant. In accordance with the study questions and hypotheses, the results of the statistical analysis were presented in tables and graphs and analysis.

Ethical considerations

Ethical considerations were taken into account, and all participants would provide informed consent before completing the questionnaire. Furthermore, the participants were informed that the data would be used for research purposes only. Participation in the study was voluntary and no personal identifiers were used to preserve the participants' confidentiality.

## Results

The demographic characteristics of the 669 participants in the study are presented in Table 1. The participants' ages were categorized as follows: 16-25, 26-35, 36-45, and >45. The highest proportion of participants (34.4%, n=230) were between the ages of 16 and 25, while the smallest proportion (13.5%, n=90) were beyond the age of 45. Regarding gender, slightly more female participants (50.5%, n=338) were present than male individuals (49.5%, n=331). The occupations of the participants were classified as student, employee, unemployed, and retired. The highest proportion of participants were employed (50.2%, n=336), while the smallest proportion retired (5.4%, n=36). The second largest group (30.8%, n=206) consisted of students, while (13.6%, n=91) were unemployed.

**Table 1:** Demographic factors of the participants (N=669)

		Count	Column (N %)
Age	16-25	230	34.4
	26-35	189	28.3
	36-45	160	23.9
	> 45	90	13.5
Gender	Male	331	49.5
	Female	338	50.5
Occupation	Student	206	30.8
	Employee	336	50.2
	Not working	91	13.6
	Retired	36	5.4

Table 2 displays the participants' CD knowledge based on their replies to knowledge questions in the self-designed online questionnaire. The table displays the number and percentage of respondents that responded “No,” “Yes,” or “I don't know” to each question. A total of (82.1%, n=549) of the total participants acknowledged having heard of CD. A total of (58.7%, n=317) of respondents had knowledge that patients with CD cannot consume gluten-containing foods. In response to the question of whether CD can occur during pregnancy or as a result of stress, (38.1%, n=255) responded “Yes,” (37.1%, n=248) responded with “I do not know,” while (24.8%, n=166) responded with “No.” Regarding whether celiac patients adhere to a rigorous diet, (40.2%, n=269) responded “No,” (32.3%, n=216) responded “Yes,” and (27.5%, n=184) said “I do not know.” On the issue of whether it is difficult for celiac patients to obtain gluten-free items, (43%, n=288) responded “Yes,” (32%, n=214) responded “No,” and (25%, n=167) responded “I do not know.”

**Table 2:** The knowledge of the participants about celiac disease based on their replies to knowledge questions

	No		Yes		I do not know	
	n	%	n	%	n	%
Have you heard about Celiac disease?	120	17.9	549	82.1	-	-
Can people with celiac disease (gluten sensitivity) eat foods that contain gluten?	317	58.7	126	23.3	97	18.0
Celiac (gluten sensitivity) occurs genetically, however, can it happen in pregnancies, and stress?	166	24.8	255	38.1	248	37.1
Travel and holidays are prohibited activities for Celiac patients (gluten sensitivity).	369	55.2	167	25.0	133	19.9
Gluten-free products in food markets are clearly labelled	221	33.0	335	50.1	113	16.9
Gluten-free products are reasonably priced at food markets in my area	329	49.2	175	26.2	165	24.7
People with Celiac disease (gluten sensitivity) are often reluctant to eat out	190	28.4	319	47.7	160	23.9
Celiac patients follow a strict diet	269	40.2	216	32.3	184	27.5
Celiac patients find it difficult to obtain gluten-free products	214	32.0	288	43.0	167	25.0
Are there a variety of different gluten-free products available on the market?	201	30.0	301	45.0	167	25.0

**Figure 1:**
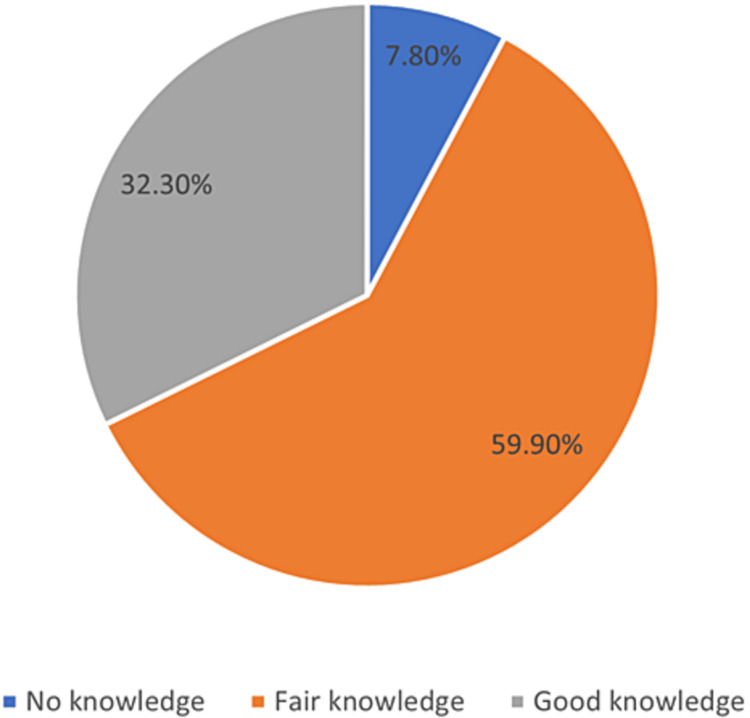
The knowledge of the participants about celiac disease

Regarding the knowledge score of the participants, (7.8%, n=52) of the individuals had no understanding of CD, (59.9%, n=401) had acceptable knowledge, and (32.3%, n=216) had excellent knowledge. The cumulative statistic indicated that (67.7%, n=453) of the participants had adequate or excellent CD knowledge (Figure 1).

The participants' psycho-social attitudes toward CD are depicted in Table 3 based on their replies to attitude questions in the self-designed online questionnaire. In response to the question of whether restaurants and cafes in the participants' area offer gluten-free options, (35.1%, n=251) responded “No,” (41.0%, n=274) said “Yes,” while (23.9%, n=160) responded “I do not know.” In response to the question of whether CD hinders a normal social life (42.5%, n=284) of respondents indicated “Yes,” (36.0%, n=241) said “No,” while (21.5%, n=144) responded “I do not know.”

**Table 3:** Attitude of the participants toward celiac disease

	No		Yes		I do not know	
	n	%	n	%	n	%
Restaurants and cafes around my area serve gluten-free products	235	35.1	274	41.0	160	23.9
Celiac disease (gluten sensitivity) is an obstacle to a normal social life.	284	42.5	241	36.0	144	21.5
I think there is enough social awareness towards Celiac disease (gluten sensitivity) in my community	319	47.7	237	35.4	113	16.9
Is there more development towards gluten-free products in your area?	177	26.5	331	49.5	161	24.1

**Figure 2:**
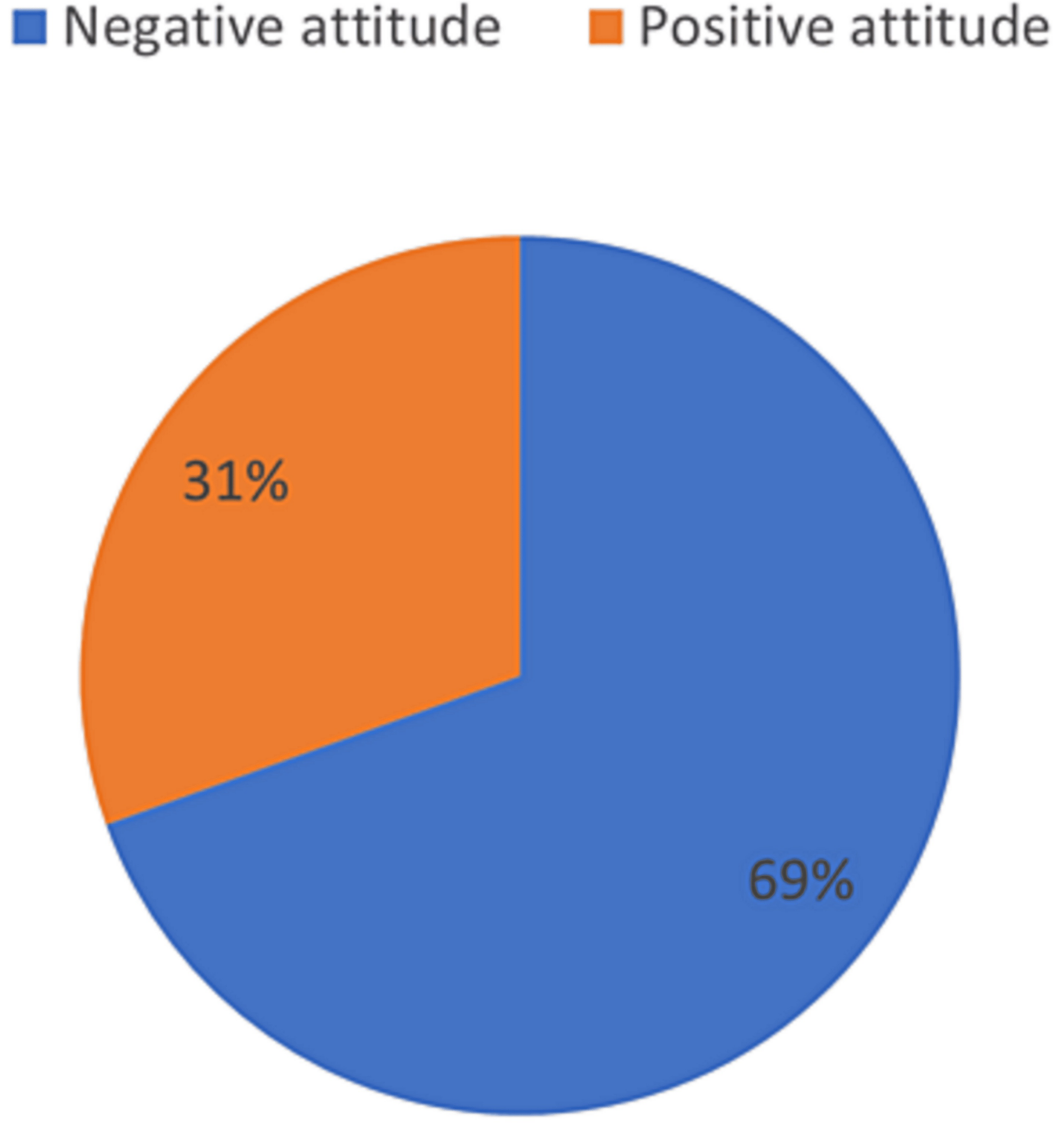
Attitude of the participants toward celiac disease

Regarding the attitude of participants toward CD (69.5%, n=465) of the individuals had a negative attitude toward CD whereas (30.5%, n=204) had a positive attitude (Figure 2).

Table 4 illustrates the association between CD knowledge and attitude and the demographic characteristics of the participants, including age, gender, and occupation. The data reveal that there is a substantial correlation between age and both CD knowledge and attitude. Participants aged 16 to 25 had the highest proportion of adequate knowledge (57%, n=131) and positive attitudes (20%, n=46) regarding CD, whereas participants aged 45 and older had the highest proportion of negative attitudes (70.0%, n=63). The correlation between age and CD knowledge (P<0.05) and attitude (P<0.05) was statistically significant. Regarding occupation, students showed the largest proportion of adequate CD knowledge (56.3%, n=116) and positive attitude (19.4%, n=40). The biggest prevalence of unfavorable attitude was found among retirees (63.9 %, n=23). The correlation between occupation and CD knowledge (P<0.05) and attitude (P<0.05) was statistically significant. There was no significant association between gender and CD knowledge (p=0.720) or attitude (p=0.244).

**Table 4:** The relation between knowledge and attitude with demographic factors of the participants

		Knowledge								Attitude				
		No knowledge		Fair knowledge		Good knowledge		P-value	Negative attitude			Positive attitude		P-value
		Count	Row N %	Count	Row N %	Count	Row N %		Count		Row N %	Count	Row N %	
Age	16-25	14	6.1	131	57.0	85	37.0	0.008*	184		80.0	46	20.0	0.000*
	26-35	12	6.3	119	63.0	58	30.7		133		70.4	56	29.6	
	36-45	10	6.3	101	63.1	49	30.6		85		53.1	75	46.9	
	> 45	16	17.8	50	55.6	24	26.7		63		70.0	27	30.0	
Gender	Male	26	7.9	203	61.3	102	30.8	0.720	237		71.6	94	28.4	0.244
	Female	26	7.7	198	58.6	114	33.7		228		67.5	110	32.5	
Occupation	Student	9	4.4	116	56.3	81	39.3	0.002*	166		80.6	40	19.4	0.000*
	Employee	24	7.1	212	63.1	100	29.8		210		62.5	126	37.5	
	Not working	13	14.3	57	62.6	21	23.1		66		72.5	25	27.5	
	Retired	6	16.7	16	44.4	14	38.9		23		63.9	13	36.1	

## Discussion

The present study sheds light on the present level of knowledge and attitudes regarding CD among the general public in Riyadh, Saudi Arabia. The findings of the current study revealed that the majority of younger-aged participants had acceptable or excellent knowledge of CD (59.9% and 32.3%, respectively). Furthermore, our study showed that a higher proportion of participants had heard about CD compared to various previous studies [12,13]. For example, a recent study conducted in Turkey reported that 43.9% of the public had never heard of CD [12]. This number is far greater than our findings which showed that only 17.9% of participants were unaware of CD. Similarly, a survey of 861 participants by Simpson et al. reported that 47% of the public in the United States (US) were aware of CD [14]. A similar level of knowledge has been reported from the United Kingdom (UK) where 44.2% of study participants were aware of the CD [15]. Our findings were much higher compared to a previous study from Saudi Arabia which reported that 48.4% of the general public had heard of CD. However, they also reported that 80.1% of participants were aware of peanut allergy and 72.4% knew about gluten sensitivity [13]. Our findings can be explained by the fact that in our questionnaire we also mentioned ‘gluten sensitivity’ along with CD. Even previous studies from Saudi Arabia have reported that more people are aware of gluten sensitivity compared to CD [13-16].

The most common misconceptions and knowledge gaps were identified by analyzing the participants' replies to the knowledge questions. For instance, one query addressed whether patients with CD could consume modest amounts of gluten-containing foods. The correct response is no, as even trace amounts of gluten can cause CD symptoms [17,18]. However, 36.7% of respondents answered incorrectly. Our findings were comparable to a previous survey which revealed that 46.1% did not know that celiacs cannot consume gluten-containing foods [12]. This lack of awareness could result in unfavorable attitudes toward individuals with CD or non-celiac gluten sensitivity who require gluten-free diets to treat their illnesses. For instance, they may encounter discrimination or stigma when attempting to buy gluten-free products or dine at restaurants. In addition, if family members of gluten-free diet-required patients with poor understanding also hold negative attitudes, this could exacerbate the difficulties these patients encounter [19].

Regarding knowledge about challenges for patients with CD, the majority of participants (83.3%) correctly identified that celiac patients encounter challenges getting gluten-free items, including restricted availability and high prices. Different previous studies have shown that patients with CD face many difficulties in identifying and acquiring gluten-free items [20,21]. One major concern that patients with CD report is anxiety and reluctance when dining out which was shared by 47.7% of the participants. This is similar to what was reported in a previous study, which found that 44.9% of participants in this study believe celiac patients experience fear and reluctance when dining out, while 32.3% believe these individuals feel constrained when participating in activities such as travel and vacations [12]. Life food restriction is a general concern that is often reported by patients with CD [22].

Various demographic parameters such as age and occupation are significantly associated with the knowledge and attitude of the participants. Younger participants and students had greater knowledge of CD and a more positive attitude compared to older respondents. This can be due to the fact that younger respondents are more likely to spend time on social media and hear about different diseases [23]. Previously, a study reported that almost 96% of celiac patients used social media to get information about their CD [24]. Although we did not find any gender-based difference in knowledge or attitude level of CD, various previous studies have reported that females are likely to be more knowledgeable compared to males [13-25]. This study provides a detailed insight into the knowledge and attitude of the general public and can help policymakers and health professionals to make effective strategies to effectively manage CD and enhance people’s knowledge.

This study had some limitations including depending on an online questionnaire that was distributed using social media which may lead to sampling bias toward younger participants, more educated participants, and neglect those who did not have an interest in dealing with these applications.

## Conclusions

This cross-sectional study shows that the majority of participants have adequate knowledge of CD; however, the majority of respondents had a negative attitude towards CD. The results also show that unfavorable attitudes towards CD must be addressed in order to encourage more social inclusion and support for those with the condition. Overall, the study highlights the significance of continued efforts to improve CD knowledge and attitudes at both the individual and societal levels in order to promote improved health outcomes and enhance the quality of life for those with the condition.
